# In vivo kinetics of early, non-random methylome and transcriptome changes induced by DNA-hypomethylating treatment in primary AML blasts

**DOI:** 10.1038/s41375-023-01876-2

**Published:** 2023-04-06

**Authors:** Gabriele Greve, Geoffroy Andrieux, Pascal Schlosser, Nadja Blagitko-Dorfs, Usama-Ur Rehman, Tobias Ma, Dietmar Pfeifer, Gerhard Heil, Andreas Neubauer, Jürgen Krauter, Michael Heuser, Helmut R. Salih, Konstanze Döhner, Hartmut Döhner, Björn Hackanson, Melanie Boerries, Michael Lübbert

**Affiliations:** 1 Institute of Genetic Epidemiology, Faculty of Medicine and Medical Center - University of Freiburg, Freiburg, Germany; 2 Institute of Medical Bioinformatics and Systems Medicine, Medical Center-University of Freiburg, Faculty of Medicine, University of Freiburg, Freiburg, Germany; 3 Department of Epidemiology, Johns Hopkins Bloomberg School of Public Health, Baltimore, MD USA; 4 Institute for Immunodeficiency, Center for Chronic Immunodeficiency (CCI), Medical Center - University of Freiburg, Freiburg, Germany; 5 Department of Medicine I, Medical Center - University of Freiburg, Faculty of Medicine, University of Freiburg, Freiburg, Germany; 6 Department of Internal Medicine V, Klinikum Lüdenscheid, Lüdenscheid, Germany; 7 Philipps University Marburg, and University Hospital Giessen and Marburg, Marburg, Germany; 8 Department of Hematology and Oncology, Klinikum Braunschweig, Braunschweig, Germany; 9 Department of Hematology, Hemostasis, Oncology and Stem Cell Transplantation, Hannover Medical School, 30625 Hannover, Germany; 10 Department of Hematology and Oncology, Eberhard Karls University Tübingen, Tübingen, Germany; 11 Department of Internal Medicine III, University Hospital of Ulm, Ulm, Germany; 12 Department of Hematology/Oncology, University Medical Center Augsburg, Augsburg, Germany; 13 German Cancer Consortium (DKTK), Partner site Freiburg; and German Cancer Research Center (DKFZ), Heidelberg, Germany

**Keywords:** Translational research, Acute myeloid leukaemia

## Abstract

Despite routine use of DNA-hypomethylating agents (HMAs) in AML/MDS therapy, their mechanisms of action are not yet unraveled. Pleiotropic effects of HMAs include global methylome and transcriptome changes. We asked whether in blasts and T-cells from AML patients HMA-induced in vivo demethylation and remethylation occur randomly or non-randomly, and whether gene demethylation is associated with gene induction. Peripheral blood AML blasts from patients receiving decitabine (20 mg/m^2^ day 1–5) were serially isolated for methylome analyses (days 0, 8 and 15, *n* = 28) and methylome-plus-transcriptome analyses (days 0 and 8, *n* = 23), respectively. T-cells were isolated for methylome analyses (days 0 and 8; *n* = 16). We noted massive, non-random demethylation at day 8, which was variable between patients. In contrast, T-cells disclosed a thousand-fold lesser, random demethylation, indicating selectivity of the demethylation for the malignant blasts. The integrative analysis of DNA demethylation and transcript induction revealed 87 genes displaying a significant inverse correlation, e.g. the tumor suppressor gene *IFI27*, whose derepression was validated in two AML cell lines. These results support HMA-induced, non-random early in vivo demethylation events in AML blasts associated with gene induction. Larger patient cohorts are needed to determine whether a demethylation signature may be predictive for response to this treatment.

## Introduction

Hypomethylating agents (HMAs) have become the treatment backbone of acute myeloid leukemia (AML) and myelodysplastic syndrome (MDS) patients unfit for intensive induction chemotherapy. Their potency lies in their DNA-demethylating properties, not found in the - structurally closely related - cytidine analogue cytarabine. Their mechanism of action is discussed to be promoter demethylation of tumor suppressor genes (TSGs) that became heavily methylated and thus aberrantly silenced during leukemogenesis. This has already been broadly studied in cell line models where 5-aza-2′-deoxycytidine (decitabine, DAC) and 5-azacytidine (azacitidine, AZA) were shown to induce TSGs important for cell cycle regulation, e.g. p15/CDKN2B and p16/CDKN2A [1], differentiation (e.g. TNF alpha) [2] or apoptosis (e.g. DAPK1, BCL2L10) [3]. In addition, recent studies suggested an additional mechanism of action in which DAC and AZA induced immune responses, either by the activation of endogenous retrovirus-derived dsRNA [4–6], the expression of immunogenic Cancer/testis antigens [7] or immune-checkpoint blocking molecules [8].

Compared to standard induction chemotherapy, single-agent HMA treatment demands longer time to best response, hence requiring more treatment cycles. Therefore it is not unusual that some patients need at least 46 HMA cycles to achieve objective responses to treatment [9–11]. To date, only very few markers exist that can robustly predict an early response to HMA treatment [12, 13], e.g. *TP53* lesions (with variable predictive power across different studies and patient cohorts [14–16]). Therefore, early molecular markers, both pre-therapeutic (determined at baseline) and dynamic (determined during treatment) are needed to identify patients who will benefit from continued HMA therapy and those who will not.

A potential HMA demethylation signature, and specifically the question whether demethylation occurs randomly or non-randomly, was studied in vitro and in vivo, e.g. in colorectal cancer where HMA treatment preferentially demethylated highly methylated non-CpG-island regions, without targeting regions bound by transcription factors and PRC2 components [17]. Another approach to uncover predictive markers is by correlating global methylation and gene expression changes. This has also been studied in cell line models, and validated by TCGA data sets [18], but only very few studies have addressed the global effects of HMAs in primary blasts serially isolated from AML/MDS patients undergoing treatment with AZA [19] or DAC [20–22]. However, no robust correlation between global demethylation and transcriptome changes could be determined yet in vivo. This was due to the high heterogeneity between patients regarding genotype (also with a high prevalence of mutations in epigenome-modifying genes), karyotype as well as baseline hypermethylation. In addition, most of these analyses have been performed on unsorted mononuclear cells (thus including T and B lymphocytes) and not on purified leukemic myeloblasts of patients treated with an HMA; also these studies did not include integrated, global methylome and transcriptome approaches.

We, therefore, conducted a prospective, genome-wide integrated analysis of methylome and transcriptome changes in purified AML blasts serially isolated from HMA-treated patients (DECIDER trial, NCT00867672) [23], hypothesizing that both random and non-random effects of the HMA may be observed in vivo.

## Material & methods

### Patients

Serial peripheral blood samples were obtained from a total of 43 newly diagnosed AML patients randomized into the DECIDER phase II trial (Decitabine/DAC treatment, 20 mg/m^2^ intravenous 1 h infusion over 5 days, with add-on drugs valproic acid and/or all-*trans* retinoic acid [ATRA] added at day 6) [23]. Of those, preparations of 28 patients yielded sufficient numbers of purified blood blasts at 3 time points (days 0, 8 and 15 from DAC treatment start) to allow a sequential analysis of matched samples (Supplementary Fig. 1; patients’ baseline characteristics are given in Table 1).

**Table 1:** Patient baseline characteristics.

Patients, *n*		43
Age, median (range)		75 [48–92]
Sex, *n* (%)	male	27 [63]
	female	16 (37)
WBC 10^3^/µl (range)		9.6 (1.1–97.5)
BM blasts, % (range)		59.0 (4.3–94.0)
Treatment cycles, median (range)		2 (1–15)
ELN 2010 risk group, *n* (%)	favourable	2 (5)
	intermediate-I	14 (33)
	intermediate-II	11 (26)
	adverse	16 (37)
Mutations, *n* (%)	*TET2*	13 (30)
	*TP53*	8 (19)
	*DNMT3A*	6 (14)

*WBC* white blood cell count, *BM* bone marrow, *ELN* European LeukemiaNet.

See further details in the Supplementary Methods.

### Methylation arrays

Genomic DNA was isolated with the DNeasy Blood & Tissue Kit (Qiagen, Hilden, Germany), bisulfite converted and hybridized to HumanMethylation450 BeadChip arrays (450 K arrays; Illumina) according to the manufacturer’s instructions at the DKFZ (Heidelberg, Germany) [7]. To avoid a possible batch effect, samples were randomized across and within plates.

### 450 K Normalization

Raw 450 K arrays.idat files were processed and normalized with the minfi R package using the functional normalization [24, 25]. Every single CpG was annotated with its nearest gene, based on the distance to the transcription start site (TSS). CpGs located on X and Y chromosomes, as well as CpGs on known SNP locations were removed. Finally, m- and beta value matrices were stored to allow for further analyses.

### 450 K Annotation

See in the Supplementary Methods.

### Group-wise methylation analysis

A group-wise differential methylation analysis was performed with the limma R package, using the m-value as input [26]. On blast samples, the following comparisons were performed: cycle 1 day 8 vs day 0, day 15 vs day 0 on 28 patients. Cycle 2 analysis was conducted on 6 patients of whom both cycle 1 and cycle 2 data at day 8 and day 0 were available. Finally, cycle 1 day 8 was compared to day 0 in T cell samples. The significance threshold was set to an adjusted *p*-value (Benjamini-Hochberg procedure [BH]) below 0.05 and an absolute delta beta above 0.1.

### Single sample delta beta analysis

See in the Supplementary Methods.

### Expression arrays

See in the Supplementary Methods.

### Expression array normalization

Raw GeneChip Human Gene 2.0 ST expression array.CEL files were processed with the oligo R package [27]. RNA intensity was normalized by RMA and unique Entrez IDs were selected using the highest interquartile range.

### Group-wise differentially expressed gene (DEG) analysis

DEGs between cycle 1 day 8 and day 0 were identified using the limma R package [26]. The significance threshold was set to an adjusted *p*-value (BH) below 0.05.

### RNA vs methylation: gene-wise correlation analysis

To compare methylation against mRNA-expression, we took the average beta values based on CpGs located in either promoter (TSS1500, TSS200, 5′UTR) or gene body (1stExon, ExonBnd, Body, 3′UTR) regions. These average beta values were directly compared to the normalized mRNA intensities using a gene-wise correlation analysis. Briefly, the RNA-to-methylome Spearman’s correlation coefficient was calculated across the 23 patients for every single gene independently by comparing the RNA log2 fold change (FC) and methylation delta beta between cycle 1 day 8 and day 0. Significantly anti-correlated genes were identified by extracting the *p*-value from the normal distribution and selected the ones below 0.05.

### Clinical response correlation with demethylation

See in the Supplementary Methods.

### Cell culture

The myeloid cell lines THP-1 and HL-60 were purchased from DSMZ (Braunschweig, Germany), authenticated and negatively tested for mycoplasma contamination. Cells were cultured in RPMI1640 medium (PAA laboratories, Cölbe, Germany) supplemented with 10% fetal bovine serum and 1% penicillin/streptomycin (PAA laboratories). Stock solution (1 mM) of DAC dissolved in PBS was aliquoted and stored in −80 °C freezer and was diluted prior to each treatment (3x) in fresh RPMI medium to the required concentrations. Experiments were repeated three times.

### RNA extraction, cDNA synthesis and qRT-PCR

See in the Supplementary Methods.

### Re-analysis of RNA-sequencing data

See in the Supplementary Methods.

## Results

### Massive, early in vivo demethylation in peripheral blood blasts of AML patients treated with decitabine

**Fig. 1:**
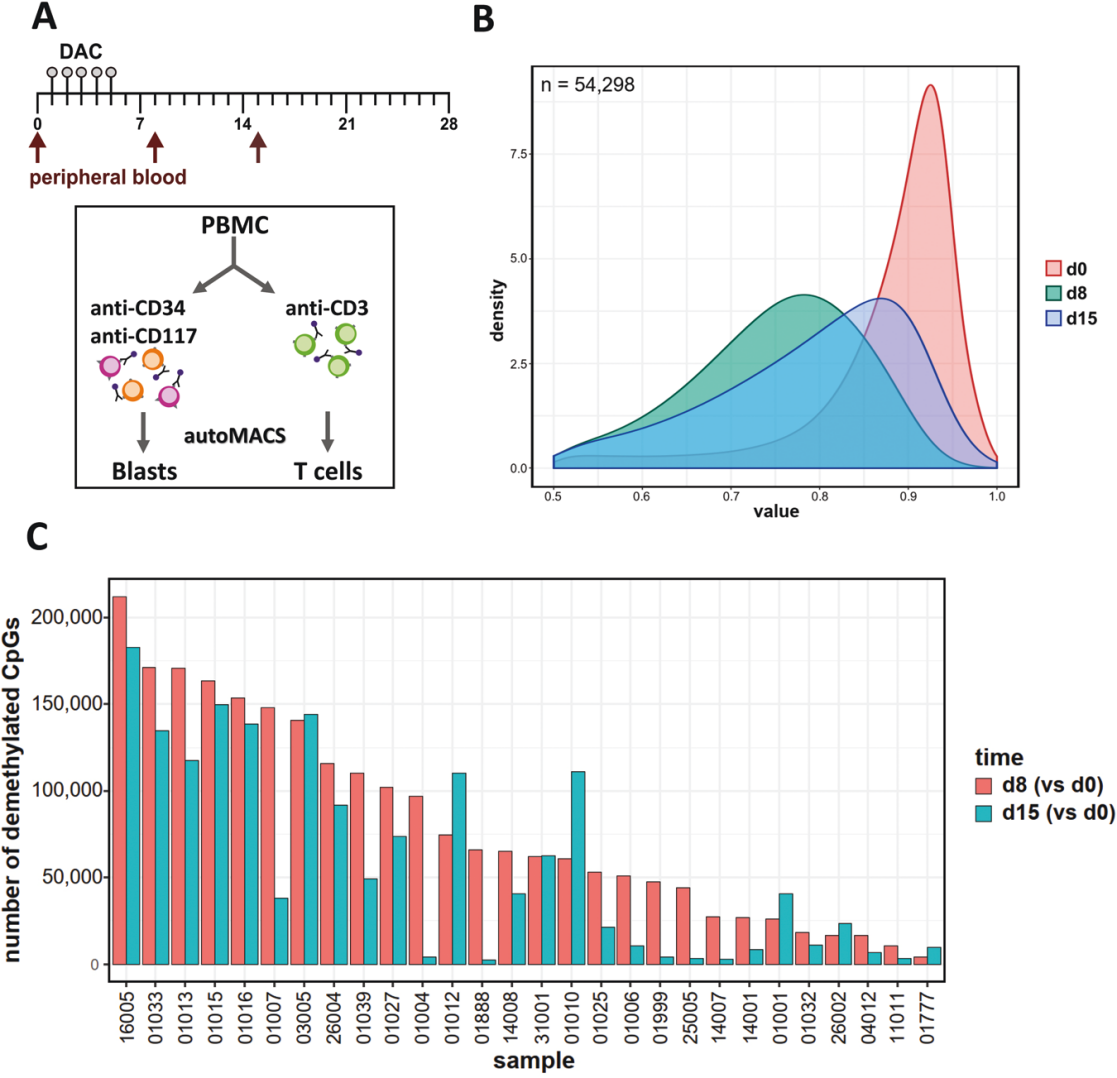
Early pronounced in vivo demethylation and partial subsequent remethylation during decitabine treatment in primary AML. **A** Schematic depiction of serial collection and purification of patient samples treated within 1 cycle of the DECIDER trial. **B** Density plot of demethylated CpGs across 28 patients at day 8 of decitabine treatment (*n* = 54,298 CpGs; green curve). 51% of these were at least partially remethylated at day 15 (blue curve). The red curve depicts the methylation status prior to treatment. (Δβ < −0.1, FDR < 0.05) **C** Number of CpGs demethylated at d8 (vs d0; red bars) and 15 (vs d0; teal bars) of cycle 1 of decitabine treatment. Methylation changes for individual patients (*n* = 28) were determined in comparison to matched day 0 samples.

We first determined the dynamic effects of DNA-hypomethylating treatment in serially sorted AML patient blasts at day 8 and 15 compared to untreated cells (Fig. 1A), hypothesizing that maximum demethylation would be observed at the day 8 time point, with partial remethylation at day 15. Confirming the results generated by Klco et al. [22], heavily methylated CpGs (defined as >50% methylated) were most affected by DAC. Across all 28 patients, at day 8 DAC significantly demethylated 11% of all CpGs represented on the array, i.e. 54,298 (of 456,281). By day 15, 51% of these became at least partially remethylated (i.e. 27,877 of 54,298, Fig. 1B). Median methylation of all CpGs (with 1.0 indicating completely methylated CpGs) was reduced from 0.90 prior to treatment to 0.75 at day 8, and increased to 0.80 at day 15. The distribution of the demethylated CpGs over genic regions was slightly skewed compared to the distribution on the 450 K array with gene body, 3′UTR and intergenic regions being overrepresented (Supplementary Fig. 2A). These demethylated CpGs were also enriched for open sea regions and repetitive elements (Supplementary Fig. 2B, C).

Next, we focused on intra-individual effects of hypomethylating treatment. In most patients we confirmed the demethylation at day 8 and its partial reversal at day 15 (Fig. 1C). Striking inter-individual differences between patients were observed in the initial demethylation at day 8, with numbers of demethylated CpGs varying between approximately 4000 and >200,000. In detail, 10 patients showed a strong demethylation response (defined as >100,000 affected CpGs), whereas 5 patients presented with a limited DAC effect on methylation (<25,000 demethylated CpGs) and 13 an intermediate response. This variability between patients was also notable on day 15, when 8 patients still displayed >100,000 demethylated CpGs, and 13 patients revealed <25,000 demethylated CpGs (6 of them <5000), with 7 disclosing an intermediate response. Hence, we could confirm and extend our previous results (Claus et al., 2013, ref. 21), with high inter-patient variability in vivo responses to hypomethylating therapy in a smaller AML cohort.

We then tested the hypothesis that the degree of demethylation and remethylation may be dependent upon the mutation profile of the individual patients regarding genes which encode epigenetically active enzymes (e.g. DNMT3A, IDH1/2, TET2, EZH2, ASXL1, BCOR, BCORL1). However, no clear pattern emerged (data not shown), which may be due to the limited numbers of patients in each subgroup. Therefore, this research question will be addressed in a much larger cohort of AML patients having received 10-day decitabine within the “inDACtion vs induction” trial EORTC 1301 (“AML21”).

We further hypothesized that the degree of demethylation could be associated with the likelihood of achieving a clinical response. We, therefore, compared both the number of demethylated CpGs and the degree of demethylation across the responder (*n* = 15) vs non-responder patient groups (*n* = 19). However, we did not observe any differences in demethylation between the responder and non-responder patients in the group-wise differential methylation analysis (Supplementary Fig. 3A), and no significant CpGs distinguishing both cohorts were found. Indeed, we could not find any CpGs that were predominantly demethylated in of the responder group (at least 80%) but not demethylated in the non-responder group (at most 20%) (Supplementary Fig. 3B).

### A common set of CpGs provides a demethylation signature of genes that are non-randomly and robustly demethylated by decitabine

**Fig. 2:**
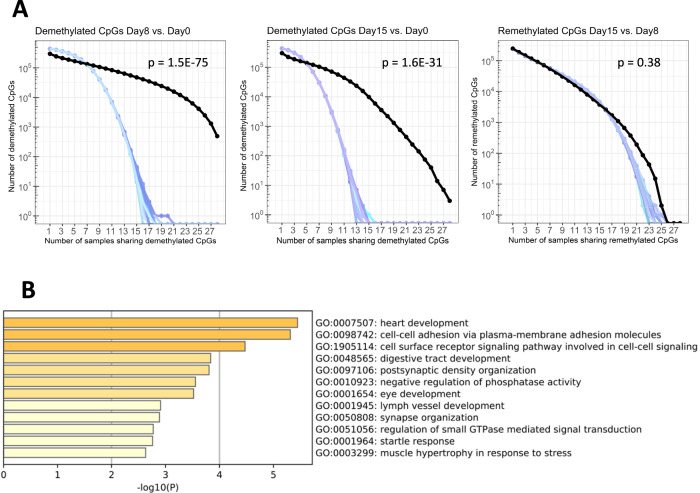
A common set of CpGs is non-randomly demethylated after 1 cycle of decitabine. **A** Compared to 1000 randomly chosen sets of CpGs (positive controls for non-specific methylation changes; random selections are shown with blue shaded curves), the number of CpGs commonly demethylated by decitabine (Δβ < −0.1, FDR < 0.05; black curve) at day 8 (vs day 0, 494 CpGs; left) and day 15 (vs day 0, 3 CpGs; middle) across up to 28 pts was much higher, indicating non-random demethylation. CpGs remethylated at day 15 (vs day 8) were similar to the random controls (right), indicating random remethylation. **B** GO-enrichment analysis of 239 genes associated with the 494 non-randomly demethylated CpGs at day 8 (vs day 0). *P*-values for enrichment are depicted in the x-axis and as a color code, with orange bars showing enriched terms with *p* > 10^−5^, yellow bars with *p* > 10^−4^ and light yellow bars with *p* > 10^−3^. Assignment to GO terms and corresponding enrichment *p*-values were generated with Metascape.

We next asked whether CpG methylation changes at different time points were random or non-random (the latter indicating a DAC-induced demethylation signature). Thus, we compared the number of commonly demethylated CpGs to 1000 randomly generated sets of CpGs as a reference control for non-specific methylation changes. We found 494 CpGs to be significantly demethylated in blasts from all patients at day 8 compared to day 0 (*p* = 1.52e^-75^; Fig. 2A, left; Supplementary Table 2), indicating non-random demethylation. By Gene Ontology (GO) terms, there was strong enrichment for genes involved in cell adhesion. This also included genes with proven or putative tumor suppressor function, several of them known to be hypermethylated in AML or other malignancies, such as *CDH13*, *FAT1* and *FAT3* (Fig. 2B). Intergenic region, open sea and repetitive elements were also overrepresented in these 494 CpGs (Supplementary Fig. 4). Also at day 15, CpGs were non-randomly demethylated (*p* = 1.61e10^-31^; Fig. 2A, middle), however, due to the aforementioned reversal of demethylation, only 3 CpGs were significantly different from the test data set (with 2 of them already targeted by DAC at day 8, Supplementary Table 3). In contrast, CpGs remethylated at day 15 (vs day 8) were similar to the random controls (*p* = 0.38; Fig. 2A, right), indicating random remethylation at this later time point.

### A common set of CpGs discloses recurrent demethylation during repeated decitabine treatment cycles

**Fig. 3:**
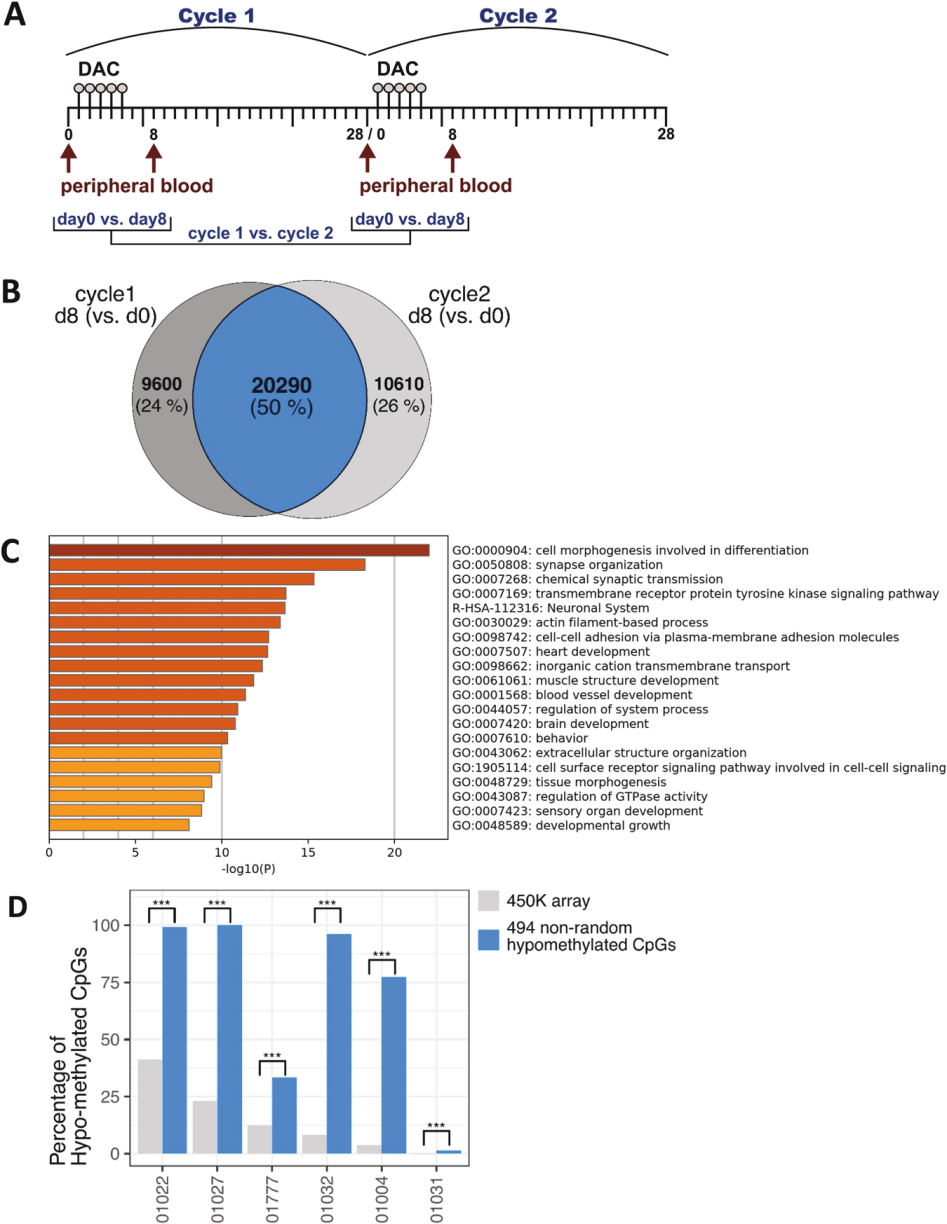
A common set of CpGs is non-randomly demethylated after 1 and 2 cycles of decitabine. **A** Schematic depiction of serial collection and comparisons of cycle 1 and cycle 2 patient blood blasts. **B** Overlapping, commonly demethylated CpGs at d8 (vs d0, FDR < 0.05, β < −0.01) across 6 patients after 1 and 2 cycles of decitabine, respectively. **C** GO-enrichment analysis of the top 2000 of the 20,290 overlapping genes demethylated at d8 (vs d0) in cycle 1 and 2. *P*-values for enrichment are depicted in the x-axis and as a color code, with brown bars showing enriched terms with *p* < 10^−20^, orange bars with *p* > 10^−20^ and yellow bars with *p* > 10^−10^. Assignment to GO terms and corresponding enrichment *p*-values were generated with Metascape. **D** Barplot showing the percentage of demethylated CpGs at day8 vs day0 cycle 2. Percentage from the whole 450 K array or the 494 CpGs signature are color-coded. Significant over-representation (Fisher’s exact test) of the 494 CpGs signature compared to the overall percentage is highlighted by “***” when *p*-value was below 0.001.

We reasoned that if in vivo DNA demethylation induced in malignant cells by HMA therapy is in part non-random, a very direct approach to test this hypothesis is the comparison of demethylating events occurring during a first vs second treatment cycle. Since HMA-based therapy often results in delayed blast clearance, we had the opportunity to study possible concordance of demethylating events in 6 patients from whom paired peripheral blood blast isolates before and after the HMA infusions could be successfully procured during both the 1st and 2nd treatment cycle (Fig. 3A). Hence, blasts were isolated at four different time points: immediately before treatment start of cycles 1 and 2, respectively, and at day 8 of both cycles, thus spanning a time interval of about 35 days.

As shown in Fig. 3B, group-wise linear-model analysis revealed 29,890 and 30,900 CpGs that were demethylated across all 6 patients during the first and second treatment cycle, respectively. Notably, 20,290 CpGs were identical to those already demethylated during the first cycle. Hence, we asked whether the 494 CpGs non-randomly demethylated in all 28 patients (at day 8 of cycle 1, as mentioned above) were represented in this subset. Indeed, 490 of 494 (99.2%) were also demethylated at day 8 of cycle 2. The top 2000 genes associated with the 20,290 demethylated CpGs showed a striking enrichment for adhesion GO terms, as well as for development/differentiation and signaling (Fig. 3C). In a complementary analysis, we asked to what extent the 494 demethylated CpGs from cycle1 were also demethylated at day 8 in single samples from cycle 2. Hence, we selected demethylated CpGs at day 8 of cycle 2, for every single patient and performed a Fisher’s exact test to see whether the 494 demethylated CpGs at cycle 1 were over-represented or not. Indeed, in all 6 patients, a strong enrichment of these 494 CpGs was found among the demethylated CpGs at cycle 2 (Fig. 3D). This sustains the non-random characteristic of the demethylation of those CpGs after DNA-hypomethylating treatment.

### Normal T cell bystander cells of AML patients undergo limited demethylation during decitabine treatment

**Fig. 4:**
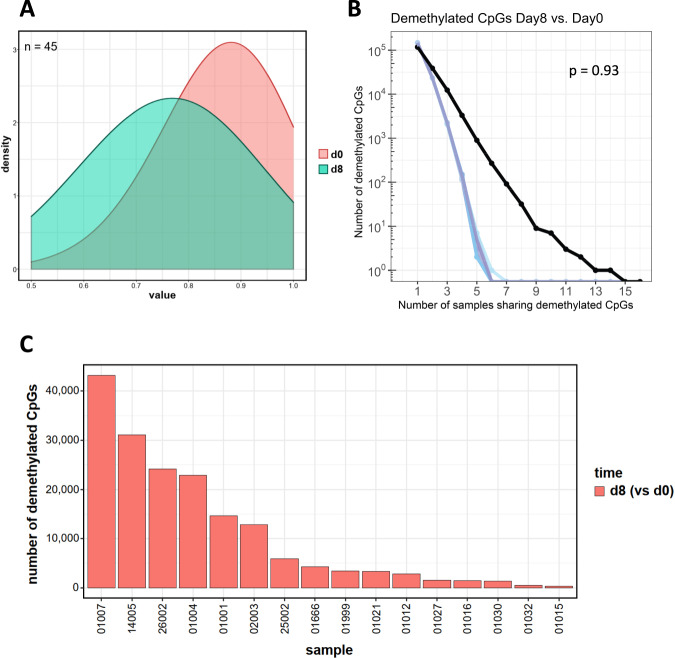
Decitabine treatment results in only limited in vivo demethylation in T cells from AML patients. **A** Density plot of demethylated CpGs in primary T cells across 16 patients at d8 of decitabine treatment (*n* = 45 CpGs; green curve). The red curve depicts the methylation status prior to treatment (d0; Δβ < −0.1, FDR < 0.05) **B** Comparison of these 45 demethylated CpGs to 1000 randomly chosen sets of T cell CpGs (positive controls for non-specific methylation changes; blue curves) indicating random demethylation (*p* = 0.932). **C** Bar plot of demethylated T cell CpGs at d8 (vs d0, FDR < 0.05, β < −0.01) after 1 cycle (red bars) of decitabine per individual patient.

Given the striking in vivo demethylating activity of decitabine in the leukemic blasts, it was of great interest to also investigate methylome changes in a non-malignant, bystander cell lineage. Therefore, we also serially isolated peripheral blood CD3-positive T cells from 16 patients at both day 0 and day 8 of decitabine treatment. Cells were subjected to methylome analyses as described above. Compared to AML blasts, the demethylating activity of the treatment was much less marked: only 45 CpGs became demethylated in a random fashion across all patients (*p* = 0.93; Fig. 4A, B; Supplementary Table 4), and the degree of demethylation was quite variable between individual patients (Fig. 4C). The limited drug effect compared to that of malignant cells may be attributable to the lower cell division rate of normal T cells compared to AML blasts; also an intrinsic resistance to uptake of this nucleoside drug can be discussed.

### In vivo promoter demethylation in AML blasts is associated with gene induction

**Fig. 5:**
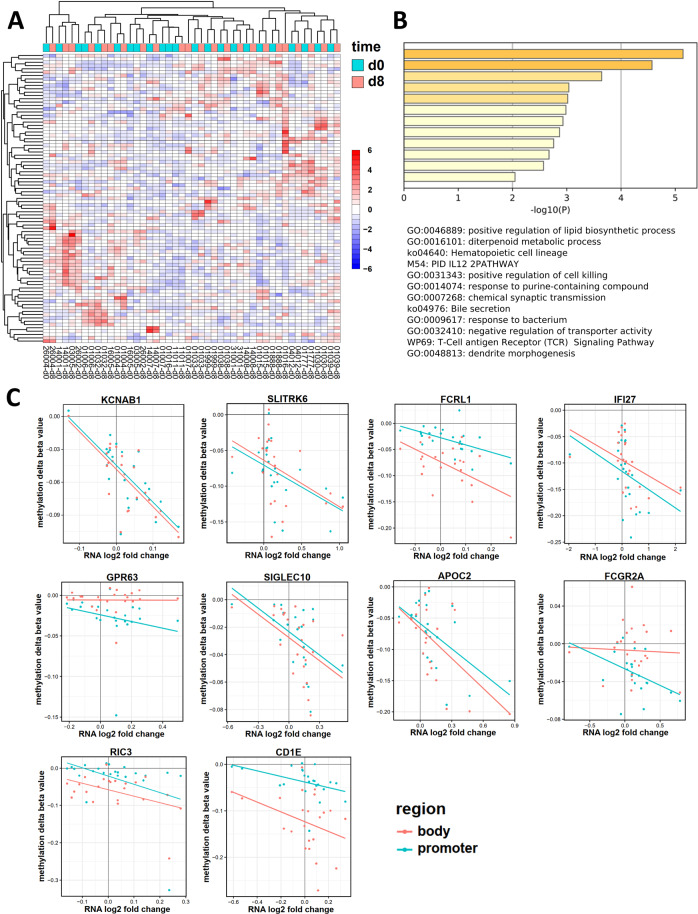
Significant in vivo correlation between promoter demethylation and gene induction by decitabine treatment. **A** Transcriptome analysis of 23 patients at d8 (vs d0, in triplicates) showed 87 genes with a significant (*p* < 0.05) anti-correlation between promoter methylation and expression after decitabine treatment across 23 patients. Non-hierarchical clustering did not indicate a specific signature. Transcriptomes were generated in technical triplicates utilizing Affymetrix expression arrays. **B** GO enrichment analysis of all 87 genes with increased expression and promoter demethylation. *P*-values for enrichment are depicted in the x-axis and as a color code, with orange bars showing enriched terms with *p* > 10^−7^, yellow bars with *p* > 10^−6^ and light yellow bars with *p* > 10^−4^ Assignment to GO terms and corresponding enrichment *p*-values were generated with Metascape. **C** Scatter plot of methylation (y-axis) and expression (x-axis) of the 10 genes with the strongest significant correlation between increased expression (indicated by positive log2 fold change) and demethylation of promoter CpGs (teal dots) and gene body CpGs (red dots). Lines depict the linear regression of the respective methylation/expression values.

To further investigate a potential decitabine-specific response signature, we next correlated demethylation and transcriptome changes. For 23 patients, the blast RNA yield was sufficient to allow for combined methylation and expression studies both prior to treatment and at day 8. Regarding expression induction upon treatment, a total of 87 genes showed a significant correlation between induction and promoter CpG demethylation (with, however, limited overall fold changes, Fig. 5A; Supplementary Table 5). GO analysis revealed enrichment for cell killing, differentiation and immune response genes (Fig. 5B). Among the genes with the most marked inverse correlation were interferon-inducible gene 27 (*IFI27*) and sialic acid binding Ig-like lectin 10 (*SIGLEC10*). They both are described to be pro-inflammatory and apoptosis-inducing and showed an increased expression (indicated by positive log2 fold change) correlating with demethylation of promoter and gene body CpGs (Fig. 5C) [28, 29].

### In vitro validation of mRNA induction upon DAC treatment of AML cell lines

We first wished to confirm in vivo induction of *IFI27* by a “reverse validation” approach using the AML cell lines THP-1 and HL-60, respectively. These cell lines both have biallelic *TP53* lesions; THP-1 also expresses the KMT2A-MLLT3 fusion resulting from the balanced chromosomal translocation (9;11)(p22;q23). They represent complementary models with respect to copy number alterations: THP-1 has predominantly copy number gains, while HL-60 has predominantly copy number losses.

**Fig. 6:**
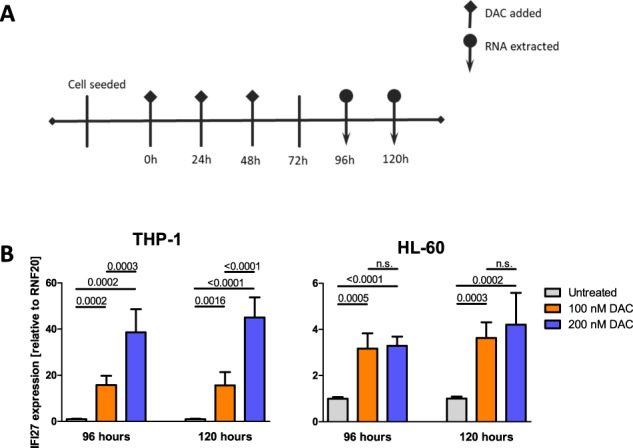
In vitro validation by qRT-PCR of IFI27 induction upon DAC treatment of two AML cell lines. **A** Treatment schedule. **B** Relative *IFI27* mRNA expression of after 96 and 120 h of DAC treatment in THP-1 (left panel) and HL-60 (right panel) cells. Relative expression was calculated against the reference housekeeping gene *RNF20*. The experiment was performed twice, each in technical replicates. Error bars depict the standard deviation (SD) of all measurements. P-values were calculated using Student’s *t-*test; paired, two-sided; n.s.=*P* > 0.05.

In the THP-1 cell line, *IFI27* expression was highly upregulated in a dose-dependent manner in response to DAC treatment (Fig. 6B, left panel). In HL-60, *IFI27* transcript levels were also significantly upregulated but the induction was less pronounced compared to THP-1 and the higher dose of DAC had no significant additional effect compared to the lower dose (Fig. 6B, right panel).

Additionally, we re-analyzed the RNA-sequencing data of the three AML cell lines UCSD-AML1, ELF-153 and U937, as published in [30, 31]. Out of the 87 genes showing inverse correlation in our patient samples, we could confirm an increased expression of the majority of genes (Supplementary Fig. 5A, B). We could also confirm in at least one cell line, respectively, the upregulation of 6 out of the 10 genes with the most marked inverse correlation (*SLITK6, GPR63, SIGLEC10, FCGR2A, APOC2* and *RIC3*; Supplementary Fig. 5C–E).

## Discussion

While DNA-hypomethylating agents have been studied for more than 50 years, their in vivo mechanisms of action are still under debate. Gene reactivation by demethylation of hypermethylated promoters is well-accepted, as is down-regulation of overexpressed genes by gene body demethylation [32]. However, only very limited data is available on the in vivo effects of HMAs on methylome and transcriptome changes in AML or MDS. Such studies are hampered by the technical and logistical challenges in procuring sufficient numbers of leukemic blood blasts at high purity, and at serial time points where additional bone aspirates would be too burdensome for the patient.

Therefore, we performed serial blast and T cell isolations from peripheral blood of AML patients receiving first-line therapy with DAC-based treatment. A key result regarding the kinetics of treatment-induced demethylation across all CpGs represented on the arrays was a high degree of variability between the 28 patients, in line with results already generated in a smaller AML cohort [21]. Therefore, we asked whether a subset of demethylated CpGs exists that is commonly targeted by this treatment across all patients, implying that demethylation is not a random event, but rather occurs at regions of the neoplastic methylome that are specifically targeted by this treatment. We provide two lines of evidence supporting this notion: Firstly, by comparing all CpGs demethylated at day 8 with 1000 randomly generated sets of CpGs, we identified 494 CpGs that were significantly demethylated in all 28 patients. In contrast, when utilizing this approach for all CpGs that became re-methylated on day 15 (vs day 8), lack of statistical significance indicated a random process. Secondly, we could demonstrate in 6 patients that during HMA rechallenge (day 8 of the second treatment cycle) a large proportion of demethylated CpGs overlapped with CpGs that had also been demethylated at day 8 of the first treatment cycle. This is, to the best of our knowledge, the first description of such reproducible in vivo targeting of hypermethylated CpGs by HMA treatment.

The “methylation rebound” occurring by day 15 of the first treatment cycle implies reversal of demethylation, as also described for *LINE-1* methylation serially analyzed in trials with guadecitabine [33, 34]. Such early remethylation points to the need for novel HMA treatment schedules with longer exposure than presently used. Here it will be of great interest to use oral HMAs, or prolonged low-dose subcutaneous exposure [35].

In 23 patients, an integrated analysis of both demethylation and mRNA expression changes at day 8 was feasible. Transcriptome changes induced in vivo by HMAs are often of a much lower amplitude than methylation changes [21, 22], and the inter-individual differences between patients are more marked than therapy-induced differences [36]. Although most demethylating events induced by HMAs are not associated with direct transcriptional activation [7, 37], we demonstrate that a sizable subset of genes displayed significant upregulation of their mRNA when demethylated. These genes were highly enriched for immune response and adhesion by GO terms, including genes such as *IFI27* (encoding Interferon alpha inducible protein 27, which can act also as a tumor suppressor) and *SIGLEC10* (encoding sialic acid-binding Ig-like lectin 10) [28, 29]. IFI27 is one of the key downstream targets of interferon response to viral infections and dsRNA-mediated viral mimicry, leading to the induction of an antiviral state causing cell cycle arrest [4, 38–40]. Interestingly, the localization of demethylated CpGs in this study showed an enrichment for repetitive elements. Furthermore, IFI27 has been described to facilitate apoptosis by easing release of cytochrome c, it interacts with and activates BAX and caspases 2, 3, 6, 8 and 9 [28]. Our results show that *IFI27* mRNA expression is specifically induced by DAC in AML cell lines THP-1 and HL-60, respectively.

Among the limited number of studies addressing the demethylating effects of HMAs in vivo, to the best of our knowledge, none have determined global methylation changes in normal “bystander” cells in parallel with changes in blast methylome configurations. In this study, we provided data from 16 matched pairs of CD3-sorted T cells, noting only very limited demethylating activity at day 8. Reasons for this massively mitigated effect of HMA treatment on the normal T cells compared to the leukemic blasts may be the lower cell cycle activity of T cells compared to blasts. However, also an overall resistance of normal, untransformed cells to uptake of demethylating cytidine analogues (due to lower expression levels of azanucleoside transporters mediating DAC uptake) can be discussed. This apparent resistance of normal cells is well in line with the limited non-hematologic toxicity of HMAs. It will be of great interest to interrogate T cell subpopulations by single-cell sequencing for potential reversal of the T cell exhaustion phenotype by HMA treatment, as has been uncovered by Ghoneim et al. employing DAC as a means to modulate T cell function in a murine model of virus infection [41].

There are also limitations to our study: as anticipated, the feasibility of successfully isolating sufficient numbers of peripheral blood blasts at all 3 time points during the first treatment course was limited to a subset of the ~150 patients enrolled on the trial and participating in the translational program. Specifically, patients had to have >10% of circulating blasts at randomization and a normal or elevated WBC count, without massive blast elimination during the first 2 weeks from treatment start. These patients therefore exhibited, as expected, a higher median WBC before treatment start than the entire population (9.6 versus 4.1 10^3^/µl), had received a median of 2 treatment courses (vs 3 in the entire population), and had a CR/CRi rate of 5.7% (vs 16.0% in the entire population). Since patients often received DAC combined with either VPA, ATRA or both, we cannot exclude the possibility that these drugs might have affected the demethylating activity of DAC at day 8 from treatment start. However, at that time point dosing of either drug had only been initiated ~48 h earlier (with a low VPA starting dose before ramp-up), which also makes confounding effects on gene transcription unlikely. Importantly, the described DNA-demethylating activity of VPA is quite limited [7] and in our hands, ATRA did not induce readily detectable global demethylation, either as single agent or when combined with DAC [31].

Despite comprehensive application of different bioinformatics approaches to probe for the predictive power of the demethylation patterns generated in vivo, we were unable to define a dynamic demethylation response signature predicting a clinical response. However, the number of patients is still limited, particularly the subgroup of patients achieving a complete hematologic remission. A follow-up translational study in a much larger cohort of AML patients receiving 10-day decitabine within the randomized phase III “inDACtion vs induction” EORTC trial 1301 (“AML21”, NCT02172872) is ongoing. Furthermore, since we used expression arrays and not RNA-sequencing for the expression analyses, it was not possible to address the transcriptional induction of transposable elements, as in our recent study using AML cell lines treated with decitabine and showing a global induction of transposable element transcripts [30].

Overall, these results support a model of therapeutic activity of HMAs that is specifically targeted, in a non-random fashion, towards the malignant clone, while sparing normal cells such as T cells. The rapid in vivo remethylation in blasts, noted already at day 15 of treatment, is supportive of extended HMA treatment schedules. Finally, the integrated analysis of gene induction associated with demethylation provides additional evidence of in vivo reactivation of genes with tumor-suppressive and immunogenic properties.

## Supplementary information

Supplementary Figures

Supplementary Methods

Supplementary Table 1

Supplementary Table 2

Supplementary Table 3

Supplementary Table 4

Supplementary Table 5

## Data Availability

Expression and 450 K methylation array data are available at GEO under accession numbers GSE171053 and GSE175758, respectively. All other datasets generated and/or analyzed during the current study are available from the corresponding author on reasonable request.
