# Supplementary material for: In vivo kinetics of early, non-random methylome and transcriptome changes induced by DNA-hypomethylating treatment in primary AML blasts
Source: Leukemia. 2023 Apr 6;37(5):1018–27. doi: 10.1038/s41375-023-01876-2 (PMC10169639; doi:10.1038/s41375-023-01876-2)
Supplement: Supplementary file 1 — Supplementary Figures [file 41375_2023_1876_MOESM1_ESM.pdf]

Supplementary Figure 1

	AML blasts	T cells	analyses
cycle 1	**28 patients**<br>day 15 vs. day 8 vs. day 0	**16 patients**<br>day 8 vs. day 0	methylation
	**23 patients**<br>day 8 vs. day 0		methylation +<br>transcriptome
cycle 2	**6 patients**<br>day 8 vs. day 0		methylation

Workflow of analyses and patient samples used in this study. Methylation analyses (Illumina 450K arrays) were performed of serially sorted blasts from 28 patients (day 0, day 8 and day 15) and T cells from 16 patients. Transcriptomes (Affymetrix expression arrays) were generated of 23 (day 0 and day 8) of these patients. Methylomes of cycle 2 samples (day 0 and day 8) were analyzed of 6 patients (day 0 and day 8 of both cycles, respectively).

Supplementary Figure 2

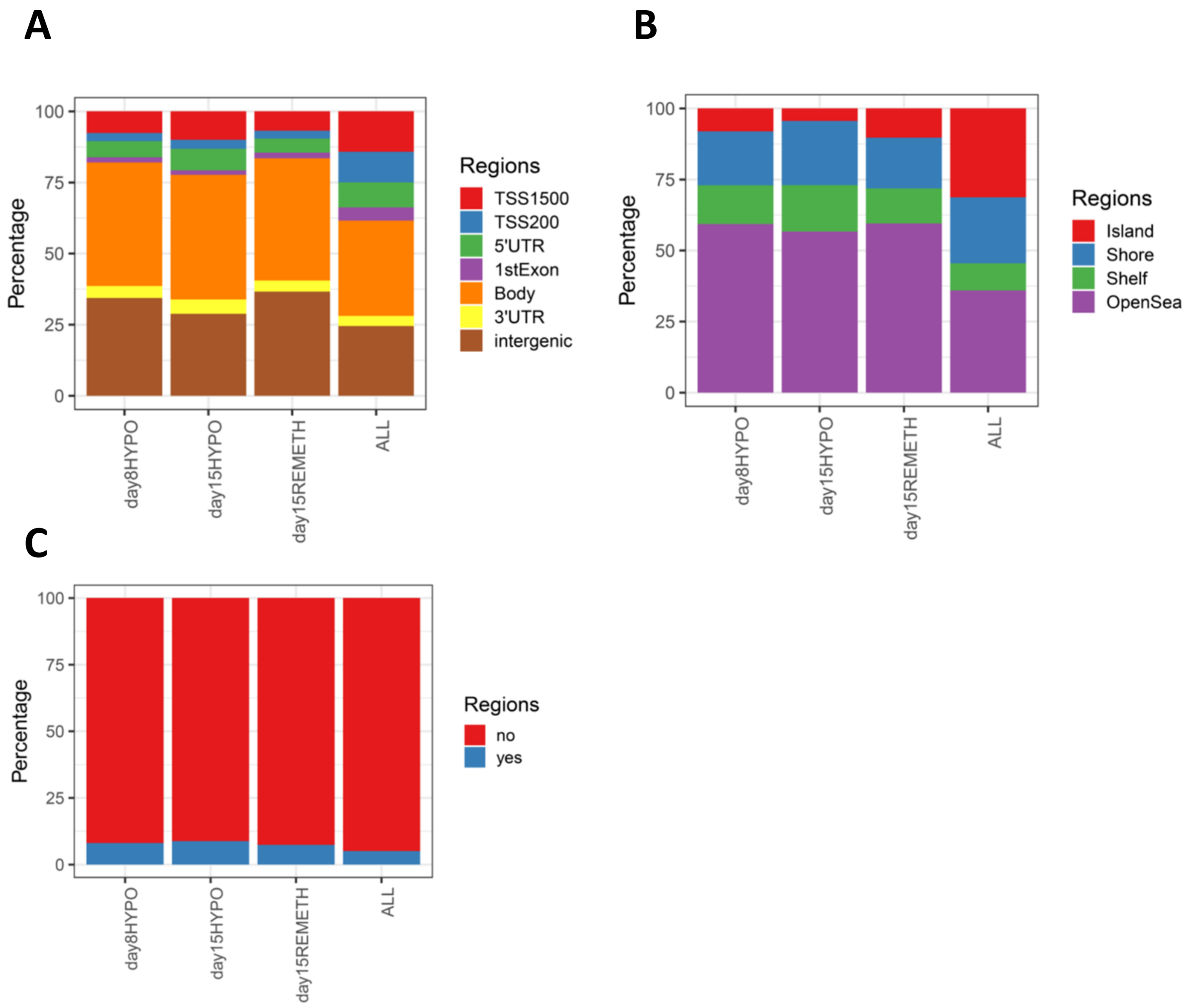

Regional distribution of CpGs significantly demethylated over all patients at day 8 ("day8HYPO"), day15 ("day15HYPO") and remethylated at day 15 ("day15REMETH") compared to all CpGs on the 450K array ("ALL"): Shown as percentages of 100% for (A) genic regions, (B) genomic regions and (C) repetitive elements.

Supplementary Figure 3

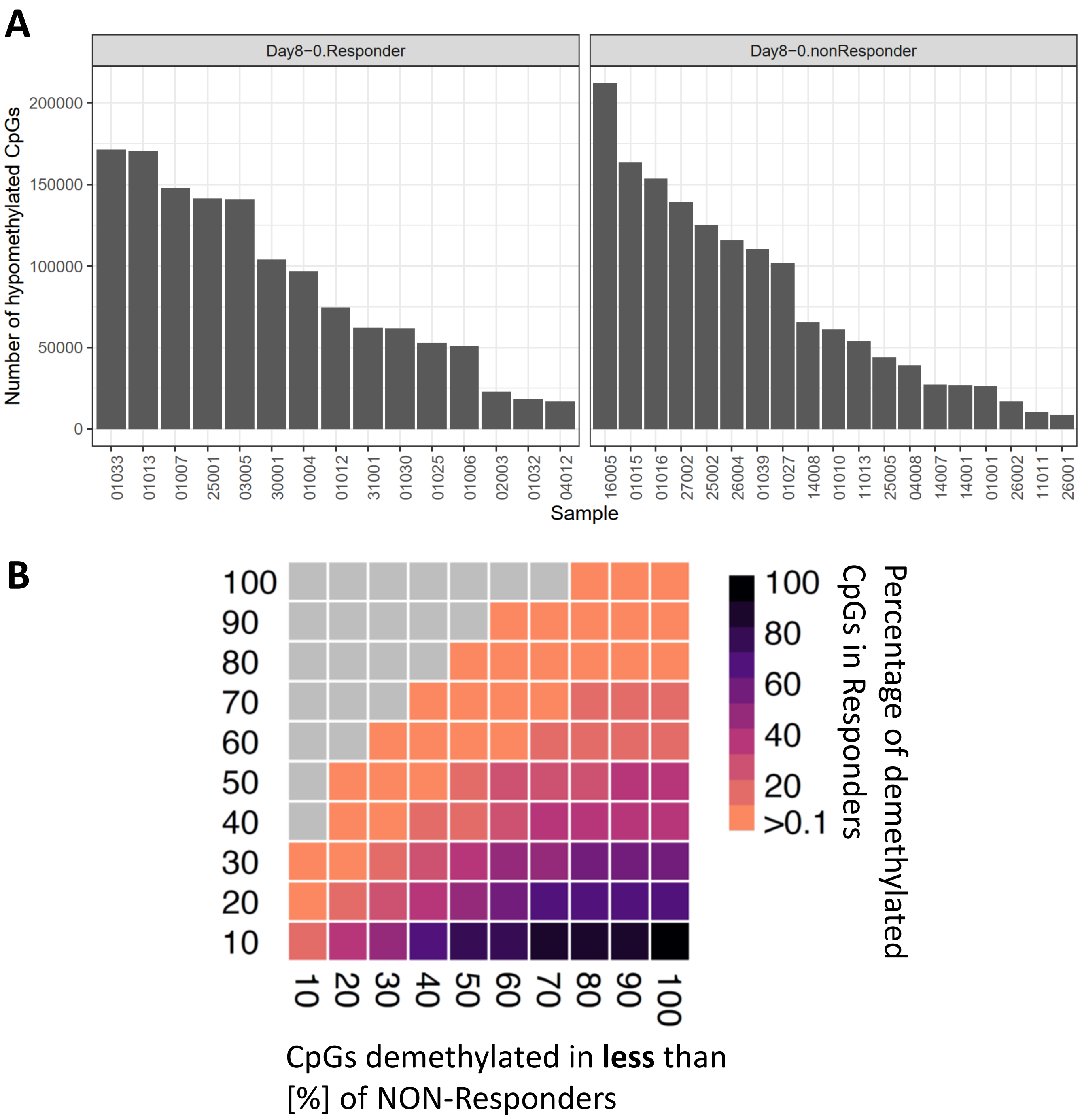

(A) Bar plot of demethylated CpGs at d8 (vs d0, FDR<0.05, β <-0.01) per individual patient. Patients were grouped for their clinical response ("responders" left, "non-responders" right). (B) Correlation matrix of CpGs demethylated in responders compared to non-reponders. The color code represents the overlap as a percentage of de-methylated CpGs in the responder group.

Supplementary Figure 4

A

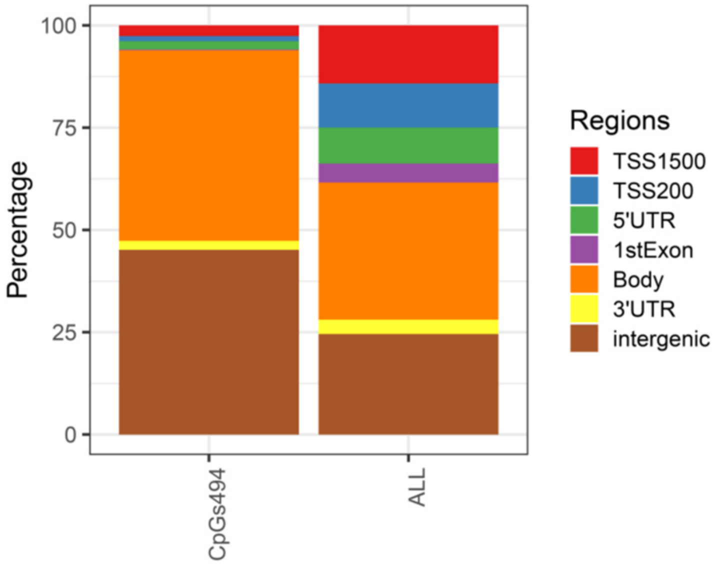

B

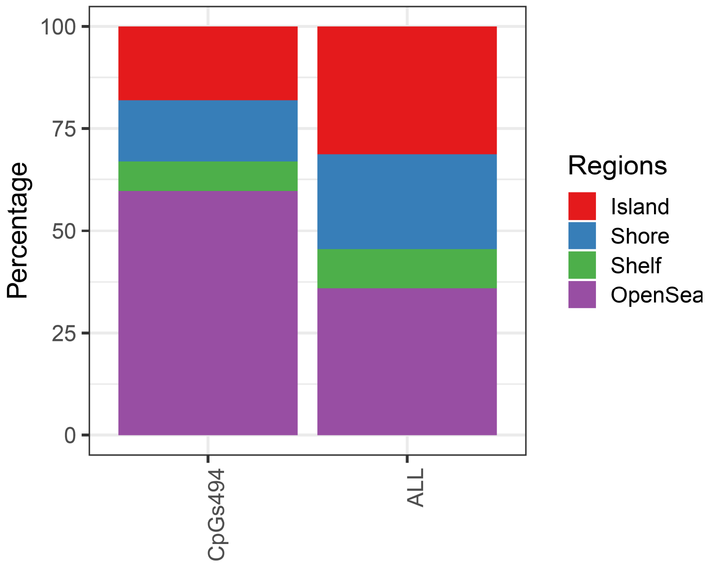

C

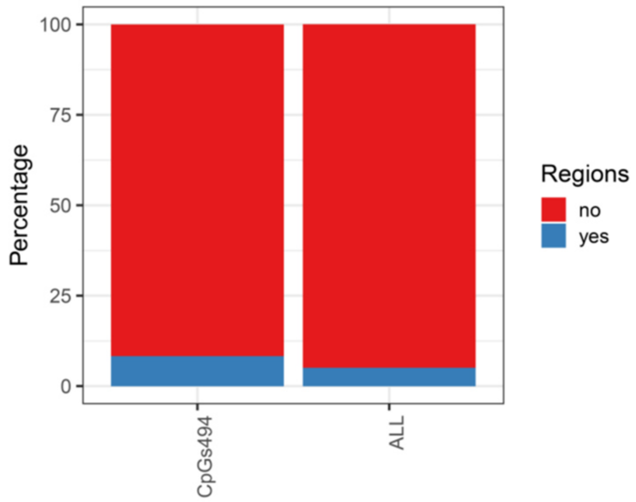

Regional distribution of the 494 non-randomly demethylated at day 8 ("CpGs494") compared to all CpGs on the 450K array ("ALL"): Shown as percentages of 100% for (A) genic regions, (B) genomic regions and (C) repetitive elements.

Supplementary Figure 5

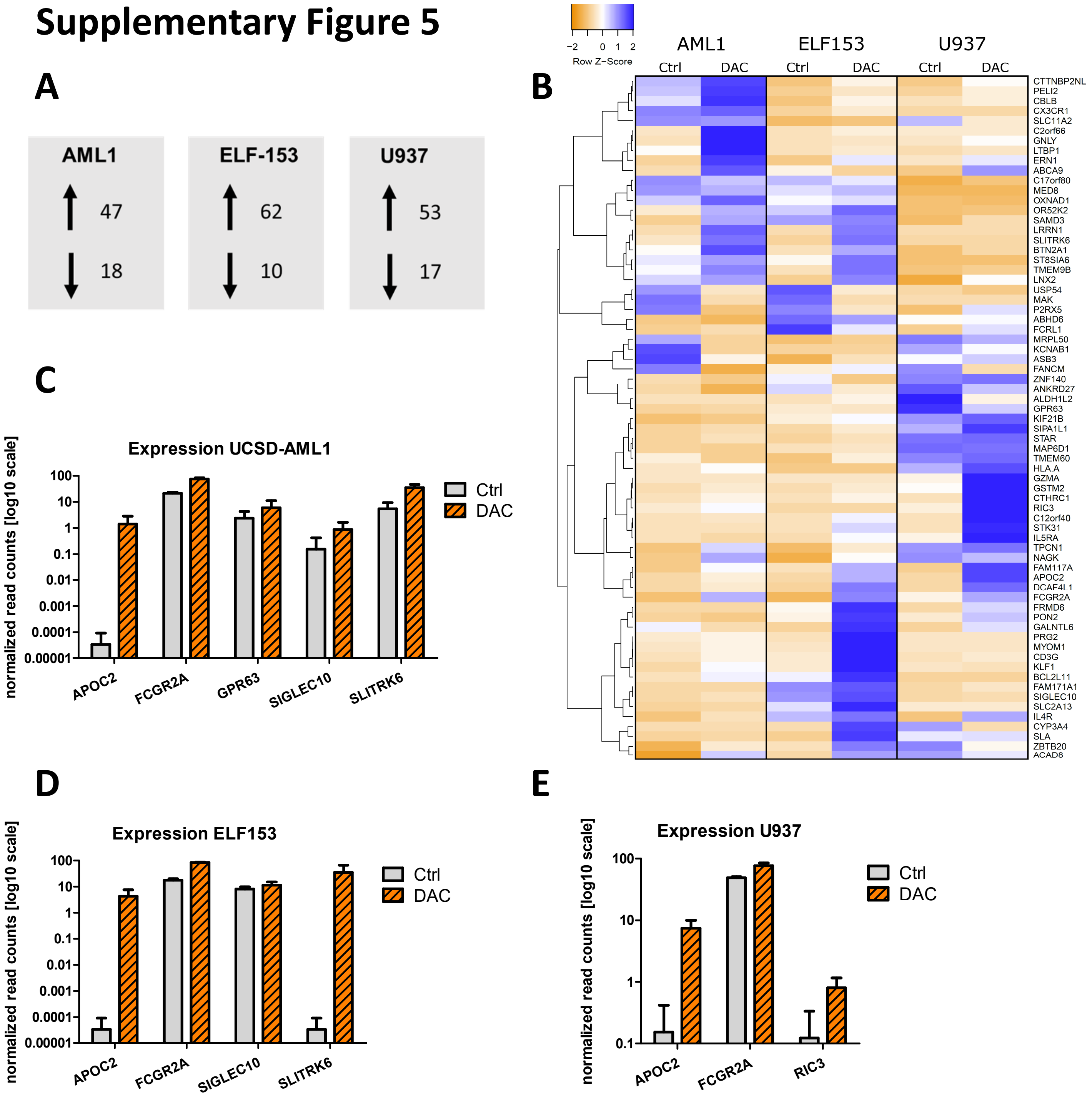

Validation of gene expression in 3 AML cell lines by RNA-seq (each condition [Ctrl = untreated, DAC] shown as mean of 3 replicates): (A) Up(🠕)- and down(🠗)regulation of all genes being anticorrelated in DECIDER patients and detected in 3 cell lines in numbers and (B) as a heatmap of Z-score converted mean normalized read counts. (C-E) Mean normalized read counts of selected genes (see Figure 5C) in 3 AML cell lines.
