# Supplementary material for: In vivo kinetics of early, non-random methylome and transcriptome changes induced by DNA-hypomethylating treatment in primary AML blasts
Source: Leukemia. 2023 Apr 6;37(5):1018–27. doi: 10.1038/s41375-023-01876-2 (PMC10169639; doi:10.1038/s41375-023-01876-2)
Supplement: Supplementary file 2 — Supplementary Methods [file 41375_2023_1876_MOESM2_ESM.pdf]

In vivo kinetics of early, non-random methylome and transcriptome changes induced by DNA-hypomethylating treatment in primary AML blasts

Gabriele Greve[1]*, Geoffroy Andrieux[2]*, Pascal Schlosser[1,3], Nadja Blagitko-Dorfs[4], Usama-Ur Rehman[5], Tobias Ma[5], Dietmar Pfeifer[5], Gerhard Heil[6], Andreas Neubauer[7], Jürgen Krauter[8], Michael Heuser[9], Helmut R Salih[10], Konstanze Döhner[11], Hartmut Döhner[11], Björn Hackanson[12], Melanie Boerries[2, 13], Michael Lübbert[5, 13]

*These authors contributed equally

Supplementary Methods

Material & Methods

Patients

One treatment cycle was scheduled for 28 days; for 6 patients (4 overlap with the 28 patients) it was possible to additionally obtain purified blood blasts (days 0 and 8) also during cycle 2. Additionally, matched T cells at cycle 1, day 0 and 8 were isolated from 16 patients (10 overlap with the 28 patients) (detailed information on patients and sample usage are given in Suppl. Fig. 1). Blasts were sorted using anti-CD34 and -CD117 and T cells with anti-CD3 MACS microbeads, respectively (median purity >90%). Patients provided written informed consent for the research use of the clinical data and biomaterial in accordance to the Declaration of Helsinki.

450K Annotation

Using the IlluminaHumanMethylation450kanno.ilmn12.hg19 annotation R package, every single CpG located on the gene body was annotated with 5'UTR, 1st exon, gene body, 3'UTR. We also annotated CpGs having a close proximity with promoter regions, i.e. transcription start site (TSS). TSS1500 and TSS200 labels define CpGs located up to 1500bp and 200bp downstream the TSS respectively. Other CpGs are considered as intergenic. The relation to CpG islands was also retrieved from the same annotation package. Each CpG is either located on CpG Islands, Shores, Shelfs or Open sea. Based on the Price et al. annotation (1), CpGs overlapping with known repetitive elements are annotated as "repetitive" whenever the entire probe was in a region of repetitive DNA. For each annotation layer (i.e. gene region, proximity to island, repetitive elements), we performed an enrichment analysis to identify over- and underrepresented regions in demethylated CpG lists compared to the 450K array distribution. Using Fisher's exact test, any regions with an odds ratio above 1 and p-value below 0.05 was considered as significantly enriched. Respectively, regions with odds ratios below 1 and p-values below 0.05 were considered as depleted.

Single sample delta beta analysis

A single-sample analysis was performed to retrieve the demethylated CpGs in every single patient. Delta beta was calculated for each patient by subtracting the beta value at day 8 from the beta value at day 0. CpGs with a delta beta below -0.1 were considered as demethylated. The p-value of the overlapping demethylated CpGs across the 28 patients in cycle 1 (6 patients in cycle 2) was assessed using a bootstrap analysis followed by an ANOVA test. To adjust for a global effect, we introduced a weighted random selection of 28 lists of CpGs. These 28 random lists contain the same number of CpGs as the measured ones. They also follow the same distribution of gene regions as the measured ones. Then the overlap across these random lists was calculated. This selection was repeated 1000 times. The number of overlapping demethylated CpGs in at least 1 to 28 samples was then compared between the

28 measured and the 28 randomized CpGs lists, generated 1000 times. Two cubic regression models were fitted to each set as described in (2). The full model captures the specific trajectory for each measured and randomized CpGs separately, whereas the reduced model specifies a single set of parameters for two sets. The goodness of the fit between the full and the reduced models was resolved by ANOVA where p-value indicates the probability that the two models are the same. Therefore, a significant p-value demonstrated a non-random overlap of demethylated CpGs.

Expression arrays

Total RNA of serially sorted blasts from 23 patients (at day 0 and 8) was isolated by TRIZOL (Thermo Fisher Scientific, Waltham, MA, USA) extraction and RNA phase was purified with the Qiagen RNeasy MinElute Cleanup Kit. Total RNA concentration was measured with the Qubit Fluorometer (Thermo Fisher Scientific) and integrity was determined by fragment analysis. RNA samples with RQN > 8.5 were further processed with the Affymetrix WT Plus kit and hybridized to GeneChip Human Gene 2.0 ST expression arrays (Affymetrix, Santa Clara, CA, USA) as described by the manufacturer (3). To avoid a possible batch effect, samples were pipetted in triplicates and randomized across and within plates.

Clinical response correlation with demethylation

The clinical outcome of patients (methylation data available from day 8 and day 0) treated and evaluated for response within the DECIDER trial (n=34) was scored by the response criteria applied for the entire patient data set of that trial. We grouped patients as either responders (complete remission with or without incomplete regeneration; partial remission, antileukemic effect, stable disease; n=15) or non-responders (progressive disease; n=19).

We performed differential analyses comparing responder and non-responder groups using the limma R package, performed in a similar manner as the groupwise methylation analysis comparing day 8 vs day 0.

RNA extraction, cDNA synthesis and qRT-PCR

RNA was isolated using NucleoSpin® RNA II Kit (Macherey-Nagel, Dueren, Germany), DNAse-digested and transcribed using the SuperScript Reverse Transcriptase III (Invitrogen, Thermo Fisher Scientific, Waltham, MA, USA) following the manufacturer's recommendations. Quantitative real-time PCR (qRT-PCR) was performed using the LightCycler® 480 SYBR Green I on the LightCycler® 480 Instrument (Roche Life Science, Penzberg, Germany) according to the manufacturer's instructions. Relative *IFI27* gene expression was determined using Pfaffl method on *RNF20* and *TGDS* as reference genes(4). Following *IFI27* and *TGDS* primers (written 5′ to 3′) were used: *IFI27*fw, GCAGTCACTGGGAGCAACT; *IFI27*rev, GAGGGGCAGGGAGCTAGTA; *TGDS*fw, GCCAGAGTGGAGAAGTTTATTTATGTCAGC; *TGDS*rev, AGGATTTGTAGGTTGTTTGGGTGAAGATT. *RNF20* primers were purchased from Primerdesign Ltd (Southampton, UK). P-values were calculated using Student's t-test (paired, two-sided).

Re-Analysis of RNA-sequencing data

See cell line characteristics and detailed methods in Greve et al. (5) and Meier et al. (6). All three cell lines, UCSD-AML1, ELF-153 and U937, were selected for their distinct (cyto)genetic features which closely resemble patients' molecular characteristics often seen in the clinic. Cell lines were treated with either 100 nM (UCSD-AML1 and ELF-153) or 200 nM (U937) DAC and subsequent RNA-sequencing was performed with at least 35 million reads / sample. Analysis of sequencing data was performed as described in (5,6).

References

1. Price ME, Cotton AM, Lam LL, Farré P, Emberly E, Brown CJ, et al. Additional annotation enhances potential for biologically-relevant analysis of the Illumina Infinium HumanMethylation450 BeadChip array. Epigenetics Chromatin. 2013;6:4.

2. Mar JC, Quackenbush J. Decomposition of gene expression state space trajectories. PLoS Comput Biol. 2009;5:e1000626.

3. Blagitko-Dorfs N, Schlosser P, Greve G, Pfeifer D, Meier R, Baude A, et al. Combination treatment of acute myeloid leukemia cells with DNMT and HDAC inhibitors: predominant synergistic gene downregulation associated with gene body demethylation. Leukemia. 2019;33:945–56.

4. Pfaffl MW. A new mathematical model for relative quantification in real-time RT-PCR. Nucleic Acids Res. 2001;29:45e–45.

5. Greve G, Schüler J, Grüning BA, Berberich B, Stomper J, Zimmer D, et al. Decitabine Induces Gene Derepression on Monosomic Chromosomes: In Vitro and In Vivo Effects in Adverse-Risk Cytogenetics AML. Cancer Res. 2021;81:834–46.

6. Meier R, Greve G, Zimmer D, Bresser H, Berberich B, Langova R, et al. The antileukemic activity of decitabine upon PML/RARA-negative AML blasts is supported by all-trans retinoic acid: in vitro and in vivo evidence for cooperation. Blood Cancer J. 2022;12:122.
